# Longitudinal changes in electrophysiology and widefield calcium imaging following electrode implantation

**DOI:** 10.1088/1741-2552/ada0eb

**Published:** 2024-12-27

**Authors:** Constance Robbins, James Eles, X Sally Zheng, Takashi D.Y. Kozai, X Tracy Cui, Alberto Vazquez

**Affiliations:** 1 Department of Radiology, University of Pittsburgh, 203 Lothrop St, EEI Suite 700, Pittsburgh, PA 15213, United States of America; 2 Department of Bioengineering, University of Pittsburgh, 302 Benedum Hall, 3700 O’Hara St, Pittsburgh, PA 15260, United States of America; 3 Center for Neural Basis of Cognition, University of Pittsburgh and Carnegie Mellon University, Pittsburgh, PA 15260, United States of America; 4 McGowan Institute for Regenerative Medicine, University of Pittsburgh, Pittsburgh, PA 15260, United States of America; 5 Center for Neuroscience, University of Pittsburgh, A210 Langley Hall, Pittsburgh, PA 15260, United States of America; 6 Neuroscience Institute at Carnegie Mellon University, Pittsburgh, PA 15213, United States of America

**Keywords:** electrophysiology, Widefield, calcium imaging, GCaMP, multi-modal recording

## Abstract

**Objective.:**

Intracortical microelectrode arrays often fail to deliver reliable signal quality over chronic recordings, and the effect of an implanted recording array on local neural circuits is not completely understood.

**Approach.:**

In this work we examined the degree of correlation and the spatial dependence of that relationship between widefield calcium imaging and electrophysiology in awake mice from 4 to 44 d post-implantation. Both correlation maps and spike-triggered averaging (STA) are used to characterize the relationship.

**Main results.:**

We find that calcium imaging and electrophysiological signal are highly correlated in all animals, however, spatial variability in the correlation is affected by inherent correlation in the calcium imaging signal. Some animals exhibit a high degree of apparent neuronal synchrony in the vicinity of the probe at 4 d, while more diversity of response is detected at later time points.

**Significance.:**

Degree of synchrony appears to be related to the acute injury response to the implanted electrode, with later time points displaying less synchrony. STA may be used to uncover the diverse cortical connections of spiking units.

## 1. Introduction

Intracortical microelectrode arrays are integral to the development of brain computer interfaces, as well as contributing significantly to current understanding of brain function through pre-clinical research. However, devices often fail to deliver reliable signal quality over chronic recordings, and the effect of an implanted recording array on local neural circuits is not completely understood [1, 2]. Additionally, the relationship between recorded spike trains and cortical activity on a mesoscale has not been comprehensively characterized, especially over the days following implantation.

The brain’s immune response to an implanted device is complex, involving multiple cell types and changing over time from acute injury to chronic residence [1, 3, 4]. Device insertion inevitably results in breach of the blood brain barrier [1, 4–6] and mechanical strain on neurons as tissue is displaced [3, 7]. Microglia are known to react immediately to device implantation, extending processes toward the device within the first hour [8], then transitioning to a pro-inflammatory ameboid morphology and migrating toward the site of injury [1, 4]. In addition to microglia, blood-derived macrophages are also recruited to the area and play a role in the subsequent neurodegeneration [5]. Within 24 h NG2 glia proliferate and migrate to the device location, with a significant response one week after injury [1, 9]. Astrocytes respond within one week. Over the course of multiple weeks they form a glial sheath to encapsulate the device, along with the microglia and NG2 cells [1, 4, 10]. The glial scar has an insulating effect, increasing impedance and decreasing signal quality [11]. A chronic inflammatory response also occurs that results in degeneration and loss of neurons within 100 *µ*m of the electrode [12, 13]. This neurodegeneration and the insulating effect both work to decrease recording quality of the device. There is also evidence the glial encapsulation exerts a complex modulating effect on nearby neuronal function [1, 14]. Under physiological conditions, astrocytes perform many functions, including clearance of potassium and glutamate from the extracellular space, serving to regulate excitability [15]. The brain’s acute injury response appears to involve hyperexcitability near the device, with reduced excitability observed at later time points [1, 16]. Astrocytes also play a role in regulating synchrony of neuronal networks, and increased synchrony has been observed in the first days to weeks of the injury response following electrode implantation [1].

Calcium imaging in transgenic mice offers the possibility to evaluate how the tissue changes during the days to weeks following electrode implantation. Widefield calcium imaging records the activity of populations of neurons in the cortex through fluorescence microscopy of calcium indicators such as GCaMP [17, 18]. Transgenic mouse lines expressing GCaMP produce stable expression in subpopulations of neurons (e.g. pyramidal) over time, suitable for longitudinal imaging, without damage to the expressing neurons [19, 20]. Widefield calcium imaging has been employed as a combined modality with electrophysiology [21–25]. Though lacking single cell resolution or ability to detect single spikes, widefield calcium imaging can allow for comparison of recorded spike trains with neural activity both near the probe and some distance from the probe. Calcium fluorescent signal has been shown to be correlated with single-unit [22, 24, 25] and multi-unit activity (MUA) [21, 22]. Correlation maps can be built by correlating the recorded spikes with the time course of each pixel in the image, revealing how correlation changes over the field of view. This method has shown spike trains to be correlated with regions near the electrode as well as more distant cortical regions, including the contralateral hemisphere [21, 22]. Spike-triggered averaging (STA) is an alternative method of assessing the relationship between electrophysiology and calcium imaging which involves averaging the calcium signal around the time of spiking of a single unit, yielding spatial and temporal information on the areas of activation both following and preceding spikes. This method has shown cortical neurons within the same animal having very similar areas of spike-triggered response, and more variety of response for sub-cortical neurons [23].

In this work, widefield calcium imaging of resting-state activity in awake mouse cortex was performed concurrently with recordings from an implanted microelectrode starting on the day of implantation for up to 44 d post-implantation, with measurements from each animal being compared longitudinally over time. The degree of correlation between these two measures of neural activity is assessed as well as the spatial dependence of that relationship. STA is performed to assess the regions of the cortex associated with each single-unit spike train.

Both correlation maps and STA are used to investigate the relationship between recorded spikes and cortical activity over the imaging region. *z*-score normalization of fluorescent signal is employed before STA to assess connectivity independently of the overall level of variability in the calcium image. We find that calcium imaging and electrophysiological signal are highly correlated in all animals, however, spatial variability in the correlation is affected by inherent correlation in the calcium imaging signal. Some animals exhibit a high degree of neuronal synchrony in the vicinity of the probe at early chronic time points (4–7 d) while more diversity of response is detected at later time points (14–44 d,) corresponding with the time course of recovery following implantation injury.

## 2. Methods

### 2.1. Animal model and implantation procedure

**Figure 1.**
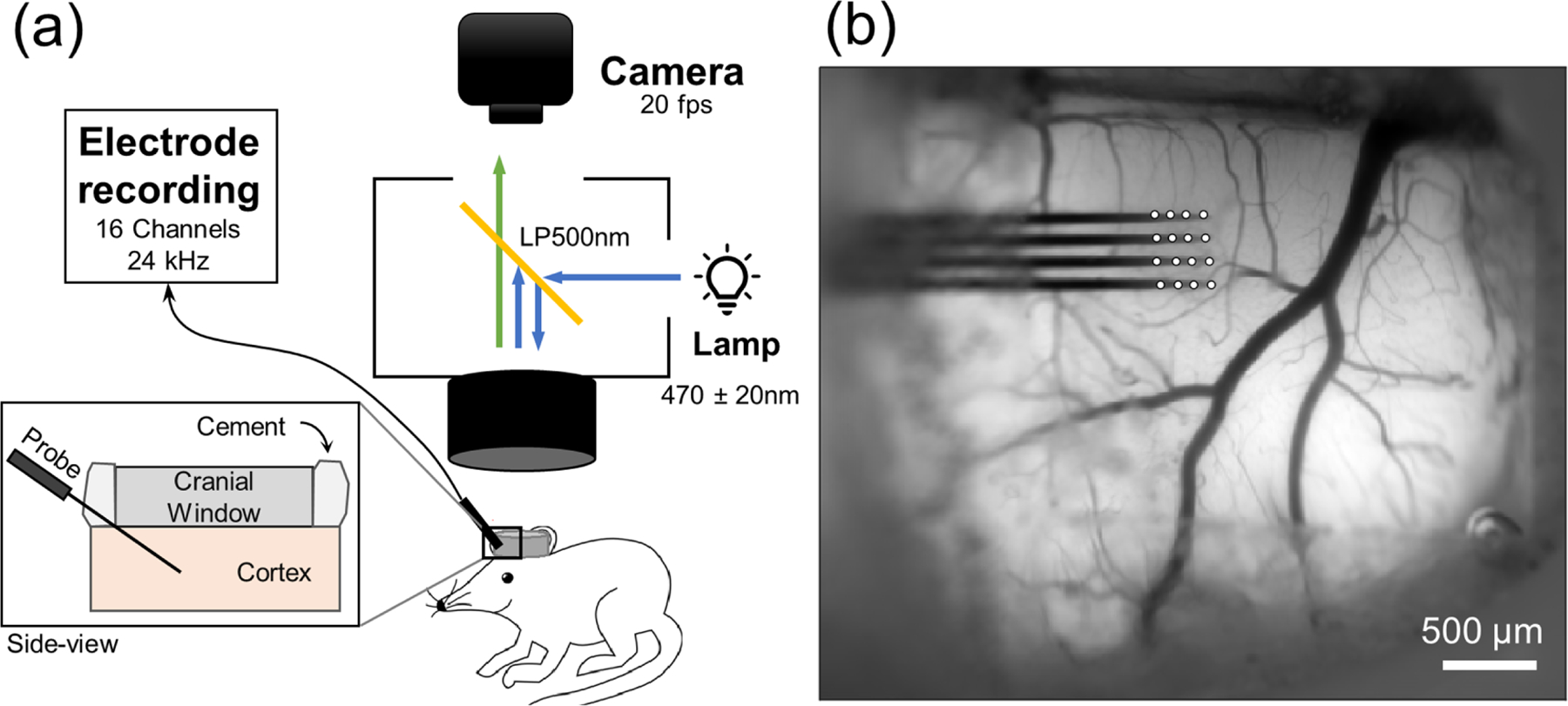
(a) Illustration of experimental setup with side-view cross-section showing probe insertion at an angle (inset.) (b) Example GCaMP image for entire field of view with white circles marking electrode recording sites.

All experiments were performed on six adult C57BL/6 J-Tg(Thy1-GCaMP6s) GP4.3Dkim/J male mice (Jackson Laboratories, Bar Harbor, ME; RRID: IMSR_JAX:024275). This line is known to express GCaMP6s [26] in a large fraction of layer 2/3 and 5 excitatory pyramidal neurons [19]. Surgery procedures were as previously described [7]. Briefly, animals were anesthetized with 75 mg kg^−1^ ketamine and 10 mg kg^−1^ xylazine via intraperitoneal injection with additional ketamine of 45 mg kg^−1^ h^−1^ injected for surgeries lasting over one hour. The scalp was shaved and resected and a craniotomy about 5 × 5 mm was performed on one hemisphere according to the procedure described in Eles *et al* [7]. A 4-shank, 16-channel silicon electrode array (NeuroNexus, Ann Arbor, MI) was implanted at a 30–35° angle using *a z*-axis Microdrive (PC-5 N, Narishige, Tokyo, Japan.) The array was fixed to the skull using dental composite. The craniotomy was covered with a glass coverslip and sealed with silicone (Kwik-Sil, World Precision Instruments, Sarasota, FL.) [27] A schematic of the experimental setup and an example widefield GCaMP image is shown in figure 1.

### 2.2. Data collection

Measurement of calcium dynamics and electrode recording was performed after craniotomy but before probe implantation (day 0) as well as on or around days 4, 7, 14, 21, 28, and 44 post-surgery. Day 0 measurements were obtained under anesthesia, while animals were awake for all following measurements. For each animal, only a subset of these imaging days is available for analysis, and in some cases, calcium imaging is available without electrophysiology. In total, 34 GCaMP measurements are analyzed, of which 17 have simultaneous electrophysiological recording available. For mice #5 and #6, calcium imaging is available but not electrophysiology. For day 21 of mouse #4 and day 14 of mouse #1, electrophysiology was not performed. For days 4 and 7 of mouse #1, electrophysiology was performed, but a technical issue resulted in saturation of the detector, and these days could not be analyzed.

Resting-state ongoing activity was imaged using single photon epifluorescence at 20 Hz (exposure time of 50 ms) for approximately 5 min with a CCD camera (CoolSnap HQ2; Photometrics, Princeton, NJ.) Animals were head-fixed under the imaging system and widefield excitation illumination of 450–490 nm was used. A longpass filter before the camera excluded wavelengths under 500 nm. Pixel size varied by imaging session, with a mean and standard deviation of 11.5 ± 0.7 *µ*m in *x* and *y*. Electrode readings were recorded with an RZ5D BioAmp Processor (Tucker-Davis Technologies, Alachua, FA) at 24 kHz. The RZ5D system digitized the trigger pulse from the camera with every frame for alignment of the data. Imaging was also performed under 620 nm illumination.

### 2.3. Calcium image processing

Motion in the widefield GCaMP imaging time-series was detected by performing sub-pixel image registration via cross-correlation with a reference frame. Images were then realigned by applying the appropriate phase shift in the frequency domain. Intensity correction was also performed to remove any signal from ambient light fluctuations or other non-neural sources of intensity change. Small regions of interests (ROIs) selected from two corners of the image (far from the craniotomy site and over the skull bone or head bar) were assumed to represent non-neural signal and were used in multiple linear regression to remove this signal from the data.

The location of the sixteen recording sites was manually identified from the 620 nm images, because they were more clearly visible in these images than in the GCaMP images. Any shift present between the 620 nm and GCaMP images was characterized as an affine transformation. The transformation was obtained using MATLAB’s ‘fitgeotrans’ function, selecting three control points in each image. This transformation was applied to the 620 nm image using the function ‘imwarp’ to align it with the GCaMP images prior to the selection of the recording site locations. In the GCaMP images, each site was assigned an ROI consisting of an ellipse of 5 pixels in the direction of the shank and 7 pixels perpendicular to the shank (approximately 45 × 64 *µ*m.) GCaMP signal was averaged over these regions to obtain a GCaMP time-trace for each recording site. For each site, a correlation map was calculated by computing the temporal correlation between the site-specific time trace and each pixel of the image. These correlation maps are referred to as GCaMP self-correlation.

The power spectral density (PSD) of the GCaMP signal within the frequency band 0.1–1.2 Hz was calculated for an elliptical ROI encompassing the 16 recording sites of the probe, and an ROI consisting of the remainder of the exposed cortex excluding large vessels, probe shanks, and a margin around the probe ROI. For each measurement, the ratio of the probe PSD to non-probe PSD is calculated.

For each imaging session, regions of the cortex with similar neural activity were grouped using *k*-means clustering. The number of clusters is set to 6 (i.e. *k* = 6), and clustering of pixels was performed based on the correlation of the time-courses of the GCaMP signal. The stability of these regions between days was evaluated for each animal. The last imaging day for each animal was used as a benchmark to compare the other imaging sessions. The clustering obtained for the benchmark imaging session was projected back onto the other imaging sessions for the same animal to evaluate the similarity of the functional connectivity of those regions over time. Registration of the benchmark cluster masks with the earlier images was accomplished by manually selecting three control points using Matlab’s ‘fitgeotrans’ function and transforming the images using Matlab’s ‘imwarp’ function. Using the benchmark cluster masks, correlation matrices were calculated for each imaging session from the average GCaMP signal within each cluster region. These correlation matrices were termed ‘functional connectivity matrices’ and the similarity of these matrices was compared longitudinally.

### 2.4. Electrophysiological signal processing

The raw electrophysiological signal was highpass filtered with a cutoff of 300 Hz. The common average reference signal (CARS) is calculated as the average of all filtered signals and is subtracted from each channel’s signal [28]. After CARS-subtraction, the correlation between channels is drastically reduced. However, in some trials, two or more channels exhibited nearly identical signals, with a very strong correlation between them. These channels were identified (correlation coefficient higher than 0.85) and were not used in any further analysis. After discarding these channels, CARS-subtraction is performed on the filtered signals using the average of only the remaining ‘good’ channels rather than all sixteen channels.

Spikes were identified by crossing of a −3 standard deviations threshold for MUA and by using a stricter threshold of −4.5 standard deviations for recording quality assessment and for single-unit activity analysis. The single-unit activity is also used for STA (described in section 2.7). Signal-to-noise ratio (SNR) was calculated for each spike according to,

(1) $$\text{SNR=}\frac{V_{\max}-V_{\min}}{2\cdot \text{RMS}},$$

where $V_{\max}$ and $V_{\min}$ represent the highest and lowest voltage occurring between 0.4 ms before to 1 ms after the threshold-crossing, and RMS is the root mean square voltage of the whole trace in which the spike was detected [29]. The magnitude of each spike was defined as the difference between $V_{\max}$ and $V_{\min}$. Total spike-yield using the −4.5 standard deviation threshold was also calculated for each channel.

MUA was calculated as the number of spikes in each time bin with the bin boundaries corresponding to the camera trigger pulses, allowing for direct comparison in the time domain between MUA and GCaMP signal. MUA was convolved with a gamma function to smooth the signal and mimic the time course of GCaMP fluorescence dynamics. Each MUA channel was paired to the GCaMP time trace of that site’s ROI (obtained in section 2.3) and the convolution kernel parameters were optimized for each pair. The convolution kernel was defined by a gamma function as,

(2) $$f\left(t\right)=C*\frac{{\left(t-t_{0}\right)}^{k-1}e^{-\left(t-t_{0}\right)/\theta }}{\text{Γ}\left(k\right)\theta^{k}},$$

where k and θ determine the shape of the distribution, $t_{0}$ is a time shift, and amplitude C represents the area under the curve of the function. Both MUA and GCaMP trace were normalized to a baseline of zero by diving by the mode value and then subtracting one. After normalization, Matlab’s ‘lsqnonlin’ function was used to minimize the root mean squared error between the GCaMP trace and MUA convolved with the gamma function kernel. Optimization of the convolution kernel was performed for each pair of GCaMP and MUA signals and the average of those gamma functions in each trial was used for further analysis.

### 2.5. Correlation of MUA and GCaMP imaging

To determine the time shift between MUA and GCaMP data, cross-correlation was performed between each MUA and its corresponding GCaMP signal. These cross-correlations were averaged, and the time shift of the maximum correlation was identified. This trial-specific shift was applied to the MUA signals before calculating the correlation of the shifted MUA and every pixel of the GCaMP image series, to produce a correlation map for each channel. Identification of the time shift was then performed for the convolved-MUA, followed by application of the shift and calculation of correlation maps between convolved MUA and GCaMP. This analysis was performed for all imaging sessions. The MUA-GCaMP correlation maps were visually compared to the corresponding GCaMP self-correlation maps, and changes in these compared over time for each animal. Two datasets did not have camera trigger pulses recorded, so the timing of frames is estimated based on the cross-correlation, and these trials were omitted from the calculation of the overall average time lag between GCaMP and MUA.

### 2.6. Spike-sorting

To obtain single-unit activity, principal component analysis (PCA) was used to group spikes according to the shape of their waveform. Though a threshold of −3 standard deviations was used for spike identification in calculating MUA, a stricter threshold was needed to resolve individual neurons by spike characteristics. After filtering and CARS-subtraction, spike times were identified based on the crossing of a −4.5 standard deviation threshold, and snippets of the signal from 0.4 ms before to 1 ms after each spike were created. For each recording site, Matlab’s ‘svd’ function was used to perform PCA on these snippets and scatter plots were created of the amplitude of the first two principal components. Visual inspection was used to determine the number of distinguishable units present in the plot. *K*-means clustering was performed with *k* = 6 for each recording site and the resulting six clusters were grouped together manually. For those recording sites that displayed distinct clusters, single unit activity (SUA) was calculated and used for the creation of spike-triggered average (STA) maps in the following section. For those without distinct clusters, MUA was used.

Firing rate for each single unit was calculated from the distribution of interspike intervals [30]. Channels for which distinct clusters could not be identified were excluded from firing rate analysis, as it is unknown if these spike trains represent a single or multiple units. Single units recording fewer than 256 spikes were also excluded, the same criteria used for STA below. For single units with greater than 256 spikes, mean firing rate, v, is defined as:

(3) $$\frac{1}{\upsilon }=\langle s\rangle =\int_{0}^{\infty }s\cdot P_{0}\left(s\right)\;\text{d}s,$$

where $P_{0}$ is the probability distribution obtained from a histogram of interspike intervals, s is the time of interspike interval in seconds, and $\langle s\rangle$ is the mean interspike interval, the inverse of the mean firing rate. Coefficient of variation, $C_{v}$, is then calculated as:

(4) $$C_{v}=\sqrt{\frac{\int_{0}^{\infty }s^{2}\cdot P_{0}\left(s\right)\;\text{d}s-{\langle s\rangle }^{2}}{{\langle s\rangle }^{2}}}$$

$C_{v}$ is a measure of how regular a spike train is, with values below one indicating more regular firing than a Poisson process, and values above one indicating more sporadic firing than a Poisson process.

### 2.7. STA

STA was used to identify the widefield neural response associated with the spiking of each identified neuron. The procedure is similar to that described in Xiao *et al* [23]. Only SUA spike trains containing 256 spikes or more were used for this analysis. Briefly, the GCaMP signal was normalized to obtain ∆*F*/*F*_0_. Snippets of GCaMP signal from −3 to 3 s around each spike time were averaged to obtain the STA response for that unit. From this response, the peak absolute value was calculated between a window of −1 to 1 s to yield a STA map (STM) representing the spatial distribution of the neural response. By using the greatest absolute value rather than the maximum value, regions of significant inhibition are also reflected in the STMs. Because the STMs for all units within a given measurement were quite similar using this method, we repeated the analysis using *z*-score normalization of the GCaMP signal, rather than ∆*F*/*F*_0_. This analysis normalizes for the variability shared between different single-unit activity STMs. The mean and standard deviation of the time-course of each pixel was used in computing the *z*-scores.

To analyze the similarity of the STMs within each measurement, STMs were thresholded to the 85th percentile and the dice similarity coefficient is calculated for each pair of STMs to yield a dice coefficient matrix. The mean of the upper triangle elements of this matrix is defined as the ‘degree of STM overlap.’

### 2.8. Statistics

Statistical tests were performed on the longitudinal comparisons derived from the GCaMP and electrophysiology signals. Because the imaging schedule varied slightly between animals, the longitudinal analysis rounds all time points to one of 4, 7, 14, 21, 28, or 44 d. A linear regression was performed for functional connectivity similarity (FCS) vs days post-implantation using MATLAB’s ‘fitlm’ function. An *F*-test is performed for goodness of fit vs a constant model to test the hypothesis that there is a non-zero slope of the trend in FCS over time. The same linear regression analysis was performed for the trends over time in SNR, average spike magnitude, number of spikes detected per channel, single unit mean firing rate, and coefficient of variation of single unit firing.

For degree of STM overlap, two-sample *t*-tests were performed to compare the degree of overlap of the distribution of day 4 with that of days 7, 14, 21, 28, and 44. For the ratio of PSD in the probe region to PSD of the non-probe region, two-sample *t*-tests were performed between the distribution of PSD ratios obtained on days 7, 14, 21, 28, and 44 d compared with the PSD ratio distribution from day 4.

## 3. Results

A total of six mice expressing GCaMP in cortical pyramidal neurons were implanted with 4 shank × 4 sites/shank NeuroNexus probes in this study. We first assessed functional connectivity using the GCaMP signal alone and assessed the stability of the connectivity over time. We then assessed the degree of correlation between the two measures of neural activity and analyzed the spatial dependence of the relationship using both correlation maps and STA.

### 3.1. GCaMP clustering and correlation

Before considering the electrophysiological data, we considered the stability of the GCaMP signal over time across the available field of view. First, we obtained functional clustering using *k-*means at each imaging time point for visual comparison of the cluster shapes over time. Following that, we took the clusters of the final imaging day and used that map as a reference to calculate the similarity of the functional connectivity matrices over time.

**Figure 2.**
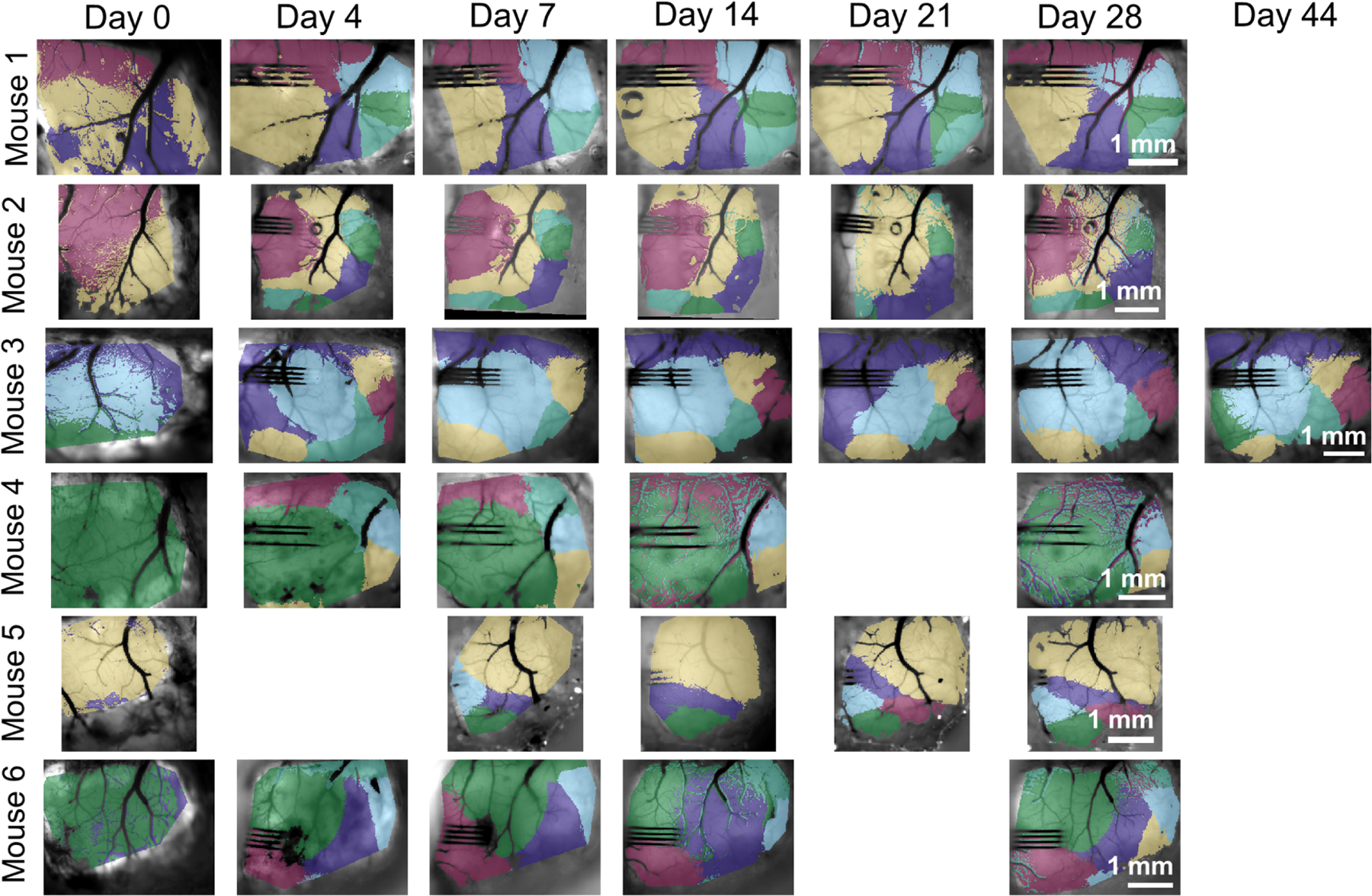
Functional clusters are obtained by *k*-means clustering of the GCaMP time-course at each pixel. Stability of this clustering is observed over time, especially when considering time points greater than 4 d post-surgery.

After *k*-means clustering was performed based on the correlation between the time-course of each pixel, the stability of these clusterings is compared between imaging days. In figure 2, color-coding of all earlier imaging days is chosen to create the best overlap with the clusters of the final (reference) day. This results in fewer than six visible clusters for some days. The appropriate affine transformation has also been applied to each of the earlier imaging days to facilitate visual comparison with the reference day.

For most animals, functional clusters appear to stabilize by around day 7. The greatest difference from the reference day is seen in the day 0 (pre-implantation) images, likely due the extremely recent craniotomy.

**Figure 3.**
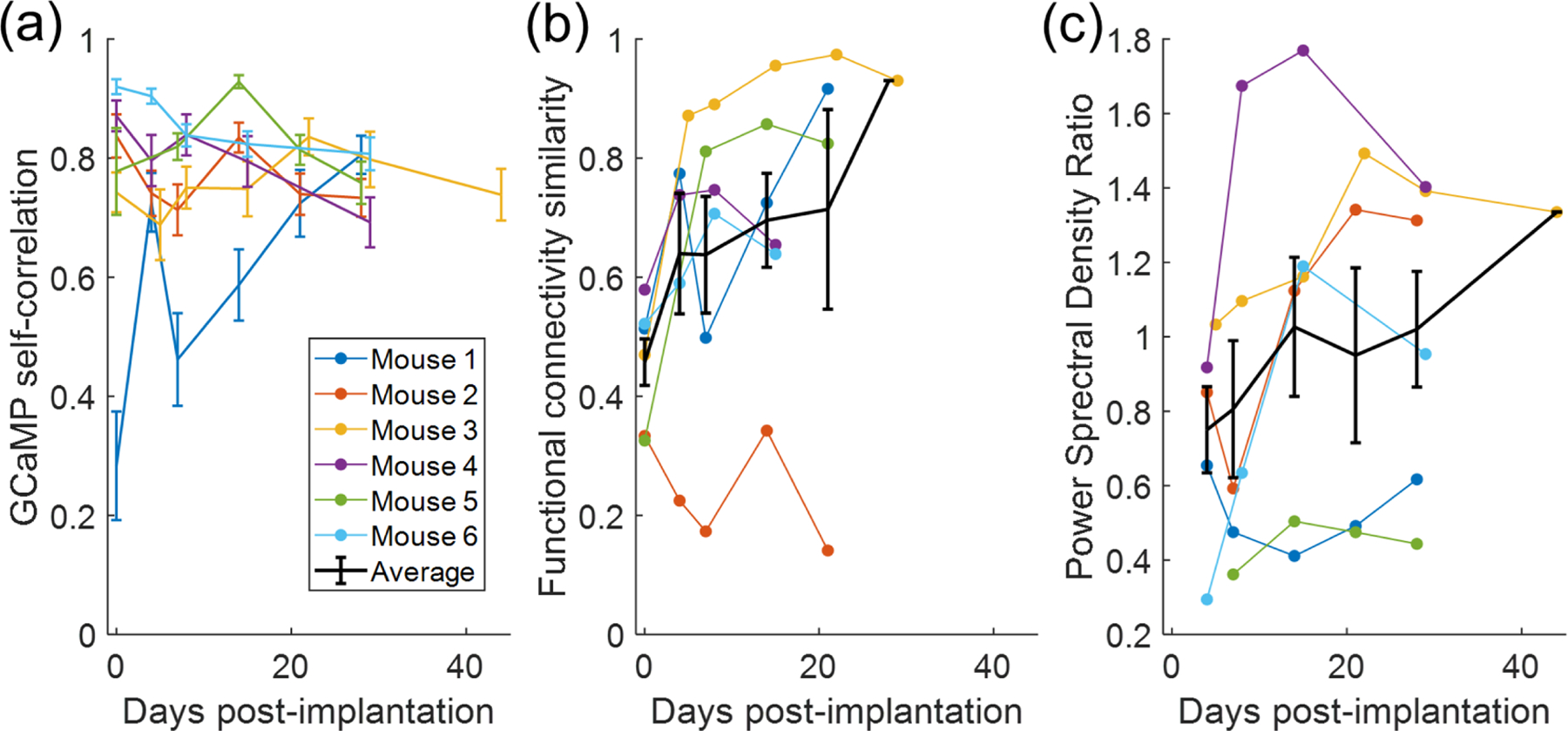
(a) Trends in correlation magnitude within the GCaMP signal over time. The mean correlation between the time-courses of the six functional clusters are plotted. (b) Similarity of the relationship between the clusters over time to the relationship on the reference day. FCS has a statically significant increase over time with *p* = 0.0408 (c) trend in the ratio of PSD of GCaMP signal at probe location to PSD in the remainder of the image. All error bars represent standard error of the mean.

Correlation matrices are calculated for the correlations between the average time course of the clusters, and these are termed functional connectivity matrices. As these matrices are symmetric about the diagonal, the average correlation magnitude of functional clusters is defined as the mean of the upper triangle elements, excluding the diagonal, of the functional connectivity matrix. This correlation magnitude over time is shown in figure 3(a). Figure 3(b) shows trends in the similarity of the functional connectivity matrices with that of the reference day. FCS is defined as the *R*^2^ of a linear fit between the corresponding elements of the two functional connectivity matrices. FCS is observed to be lowest on day 0 (pre-implantation), and to gradually increase in most mice. This is consistent with the finding that the functional clusters obtained from the pre-implantation images bear the least visual similarity with the benchmark cluster masks. Visual inspection of the post-implantation functional clusters shows consistency in the clustering over time. A linear fit was performed for the combination of all animals testing the relationship between FCS and measurement day. The slope of this fit is positive with *R*^2^ = 0.151, indicating a low proportion of the variance in FCS is explained by post-implantation day. This relationship does reach statistical significance with *p* = 0.0408.

The ratio of the PSD of GCaMP signal in the probe ROI to that of the non-probe ROI over time is shown in figure 3(c). For four animals, PSD ratio appears to increase over the course of the experiment, meaning calcium imaging activity near the probe increases relative to the rest of the image, and two animals exhibit no increase. For some animals, there is a visually distinct shadow around the probe region in the 2D map of PSD for early time points that disappears or recovers by approximately day 21. Examples are shown in supplemental figure 1. As the area of reduced PSD around the probe is most evident on day 4, the PSD ratios of later days are tested against day 4 to detect the presence of the shadow. When considering all animals, no day has a distribution statistically significantly different from that of day 4.

### 3.2. Electrophysiological signal quality

**Figure 4.**
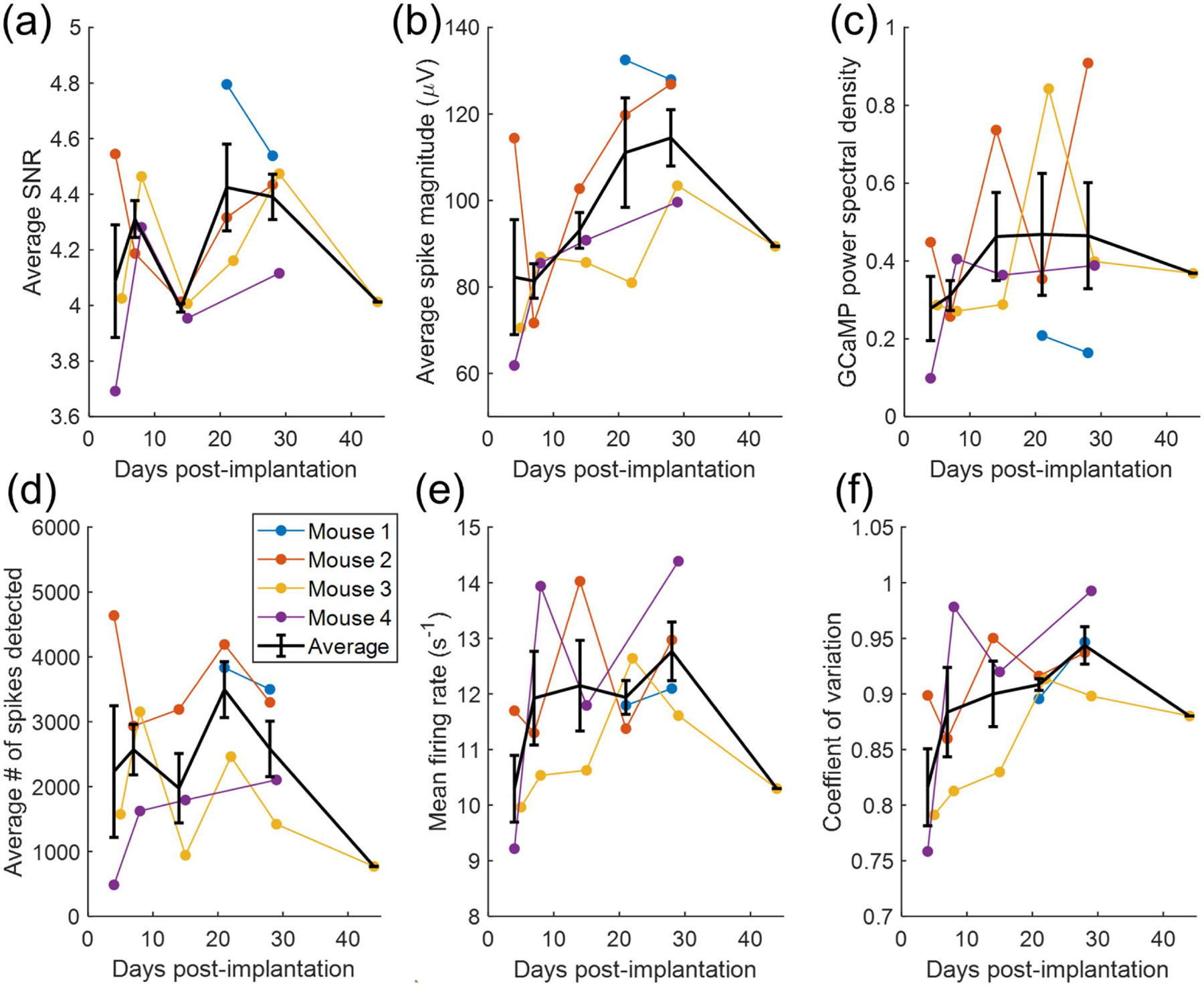
Electrophysiological recording quality compared with underlying brain activity. (a) Average SNR over time, (b) average magnitude of spikes in *µ*V. (c) Average PSD within the frequency band 0.1–1.2 Hz of the GCaMP signal in the vicinity of the recording sites, with no significant change over time (d) average spike-yield per channel does not significantly change over time, (e) mean firing rate of single units yielding more than 256 spikes, and (f) coefficient of variation (*C*_v_) of the same subset of units. *C*_v_ values below one indicate more regular firing than a Poisson process, and values above one indicate more irregular firing than a Poisson process. Both spike rate and *C*_v_ appear lowest on day 4, but this trend does not reach statistical significance.

Trends in recording quality metrics over time are shown in figure 4. Neither SNR nor spike yield show a statistically significant trend with time, with *p* = 0.51 for SNR and *p* = 0.76 for spike-yield. However, when considering fluctuations in spike-yield over time, we sought to determine whether these changes reflected differences in the underlying brain activity. The PSD of the GCaMP signal within the vicinity of the recording sites in plotted in figure 4(c). This measure represents the brain activity of the population of neurons near the recording sites but outside the detection limit. Visual inspection suggests a relationship between spike yield and PSD of the GCaMP signal, especially for mouse 3 and mouse 4. That is, days with a particularly low or high spike yield seem to correspond with low or high GCaMP activity, respectively.

Figure 4 also shows trends in the average magnitude of detected spikes and firing patterns over time. Spike magnitude appears to increase over time, but this trend does not reach statistical significance, with *p* = 0.061. The mean firing rate and coefficient of variation appear to increase over time, especially before day 21, but these trends do not reach statistical significance, with *p* = 0.423 and *p* = 0.073, respectively.

#### 3.3. Correlation between MUA and GCaMP

**Figure 5.**
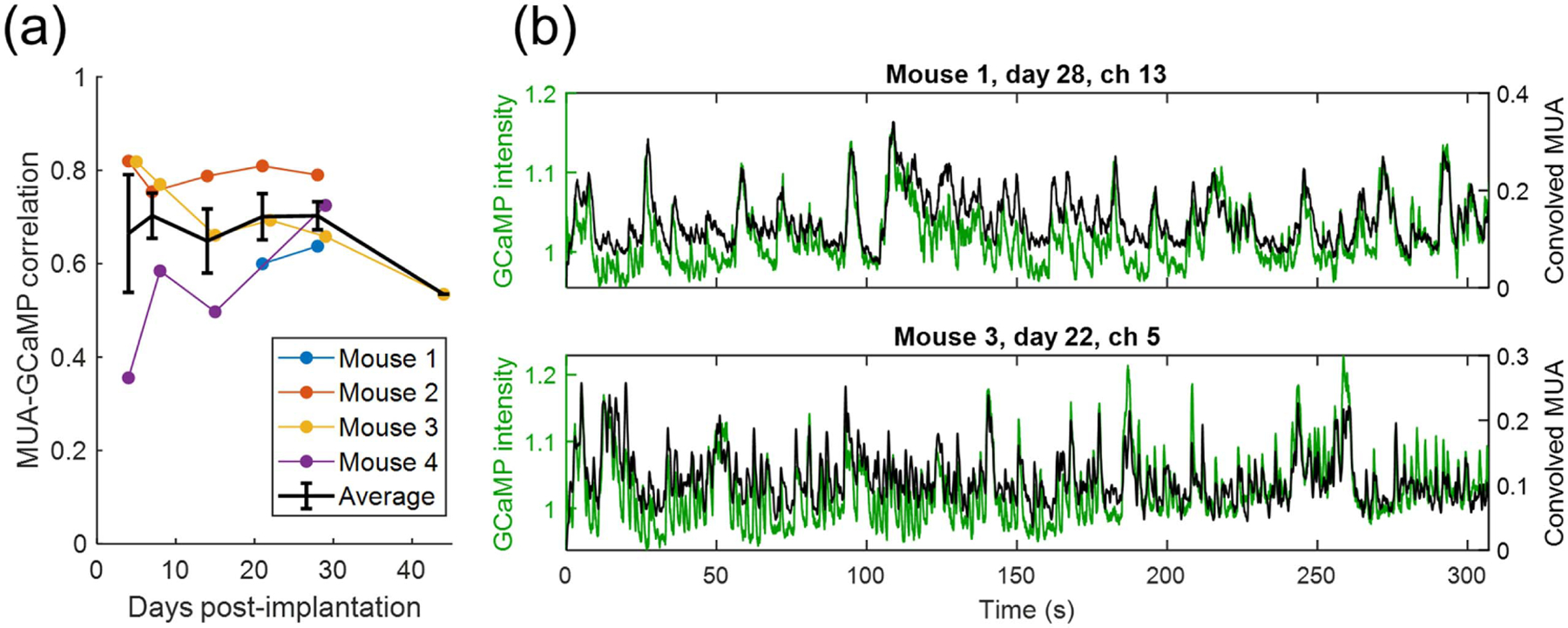
Correlation between convolved MUA and GCaMP. (a) Average correlation between convolved MUA and co-localized GCaMP signal over time, with error bars representing standard error of the mean. There is no significant change in MUA-GCaMP correlation over time. (b) Example of the relationship between convolved MUA and co-localized GCaMP for two recording sites from different animals.

Cross-correlation of MUA with its co-localized GCaMP signal was performed both with and without convolution of MUA with the gamma function kernel, as described in section 2.5. Without convolution, the peak correlation occurred with GCaMP lagging MUA by an average of 0.282 ± 0.041 s, and a mean Pearson correlation coefficient, *R*, of 0.353 ± 0.129. Convolution with the optimized gamma kernel was found to increase the strength of the correlation and decrease the time shift between the signals. With convolution, GCaMP led the MUA signal by 0.066 ± 0.099 s and a mean correlation coefficient of 0.6687 ± 0.1643. This correlation is not observed to change over time. Peak correlation of convolved MUA and GCaMP is shown in figure 5, along with two representative examples of the time domain GCaMP and convolved MUA signals for visual evaluation of the correlation. We can obtain a conservative threshold for Pearson’s *R* by considering the comparison to have at least 400 independent time points (fewer than the true number of data points to account for the smoothing effect of the GCaMP kinetics) and controlling for 16 comparisons per measurement due to the 16 recording sites, requiring *p* = 0.05/16 = 0.0312. These requirements result in a threshold for Pearson’s *R* of approximately 0.147 to be considered statistically significant. This threshold is exceeded for 86.4% of comparisons of GCaMP and raw MUA. After the convolution of MUA, the threshold for significance is met in 100% of comparisons.

**Figure 6.**
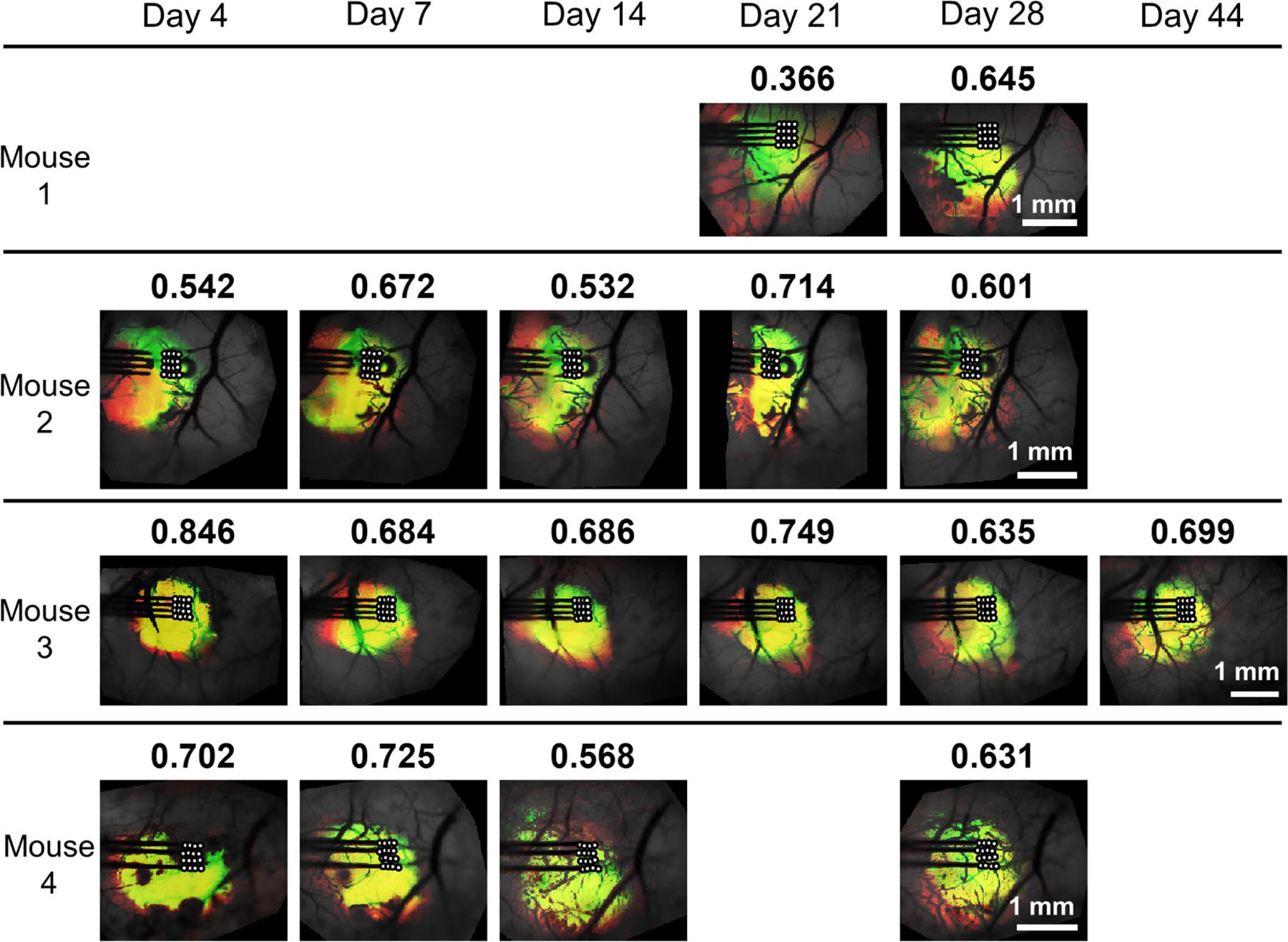
Heatmaps summarizing the comparison between the MUA correlation maps and GCaMP self-correlation maps. The thresholded MUA correlation maps for the 16 recording sites are represented with red intensity, and the thresholded GCaMP self-correlation maps for the 16 corresponding ROIs are represented with green intensity. The mean dice coefficient for all channels is shown above each image. White circles represent the recording site locations. For most cases there is considerable overlap between the MUA-GCaMP correlation and the GCaMP self-correlation regions, indicating that these areas of the cortex are experiencing shared activity and thus also have high correlation with MUA within those regions.

Correlation maps were obtained for the correlation between each recording site MUA and all pixels of the GCaMP signal, called the MUA-GCaMP correlation maps. A GCaMP self-correlation map for each recording site was calculated using the average GCaMP signal in the ROI located at recording site as the seed to correlated with the remainder of the image. Both types of correlation maps were thresholded at the 85th percentile, and the MUA correlation was compared to the GCaMP self-correlation for the corresponding recording site. Intuitively, the thresholded GCaMP self-correlation map represents a region of the cortex with shared activity to the region of the recording site. Thus, the thresholded MUA correlation map would be expected to overlap with this map if the neural activity detected by the electrode was consistent with the fluorescently recorded activity in the immediate vicinity. To examine this overlap, as well as the consistency of the correlation maps between channels, heat maps are created and shown in figure 6. The Dice coefficient between the MUA correlation map and GCaMP self-correlation map was calculated for each channel and the mean dice coefficient is displayed above each image.

A high degree of overlap is seen for most mice as well as a high consistency between recording sites. That is, the high similarity of the MUA traces produce similar correlation maps for each recording site. The exception is mouse 1 on day 21, and to a lesser extent day 28, which shows a more variation in the MUA correlation maps, visible in the heatmap as a diffuse area of dim red intensity.

### 3.4. Spike sorting and STA

In total, 35 recording sites exhibited two distinct units, 14 exhibited three distinct units, and 77 could not be distinguished into clusters. Additionally, 34 sites recorded fewer than 256 spikes, and were not included in STA analysis.

**Figure 7.**
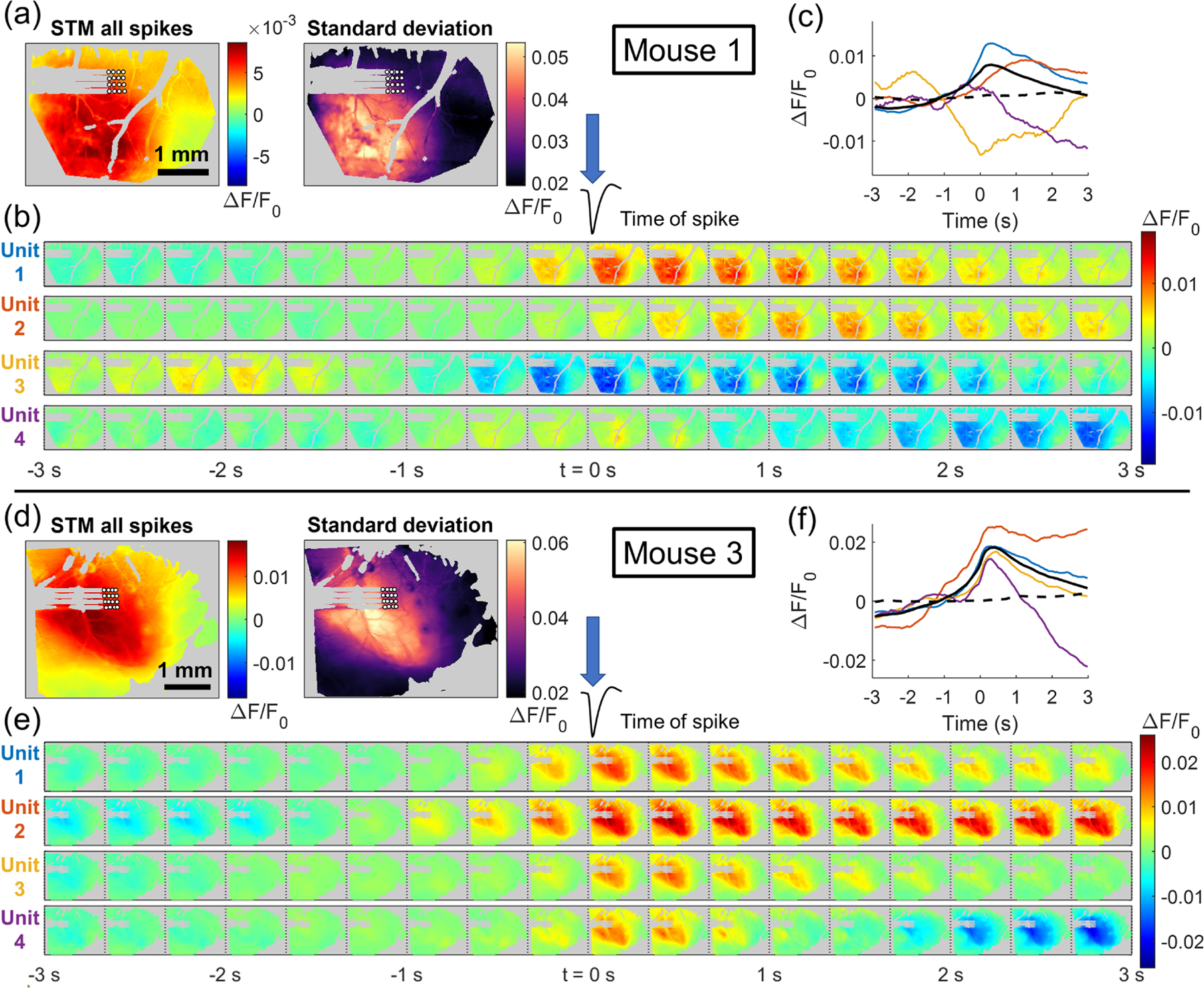
Example of spike-triggered average response for four spiking units from mouse 1, day 28 (top) and mouse 3, day 14 (bottom). (a) and (d) Comparison of spike-triggered map (STM) for all spikes and standard deviation of the time course of each pixel. (b) and (e) Example spike-triggered average responses from −3 to 3 s around the time of the spike. (c) and (f) Average time course of the spike-triggered responses within an ROI chosen from an area of high activation on the standard deviation map. The solid black line represents the combined spike-trigged response of all spikes from all channels. The dashed black line represents the spike-trigged response from randomly chosen spike times. In both animals, the STMs using all spikes and the spike-triggered responses in (b) and (e) represent the areas of highest variability in the calcium image. *Z*-score normalization is employed in further analysis to try to remove this shared effect between units.

Examples of the STA response for four neurons of two animals are shown in figure 7. Figures 7(a) and (d) show the spike-triggered maps (STMs) derived from all spikes compared to the standard deviation of the ∆*F*/*F*_0_ fluorescence signal. For both mice, the global STM closely reflects the regions of highest activity in the standard deviation maps. The STA responses from −3 to 3 s from the spike shown in figures 7(b) and (e) also display similarity to the standard deviation map, with both areas of highest activation and areas of inhibition corresponding to the areas of greatest overall variation in the GCaMP signal. The average time-course of the four STA responses are shown in figures 7(c) and (f).

**Figure 8.**
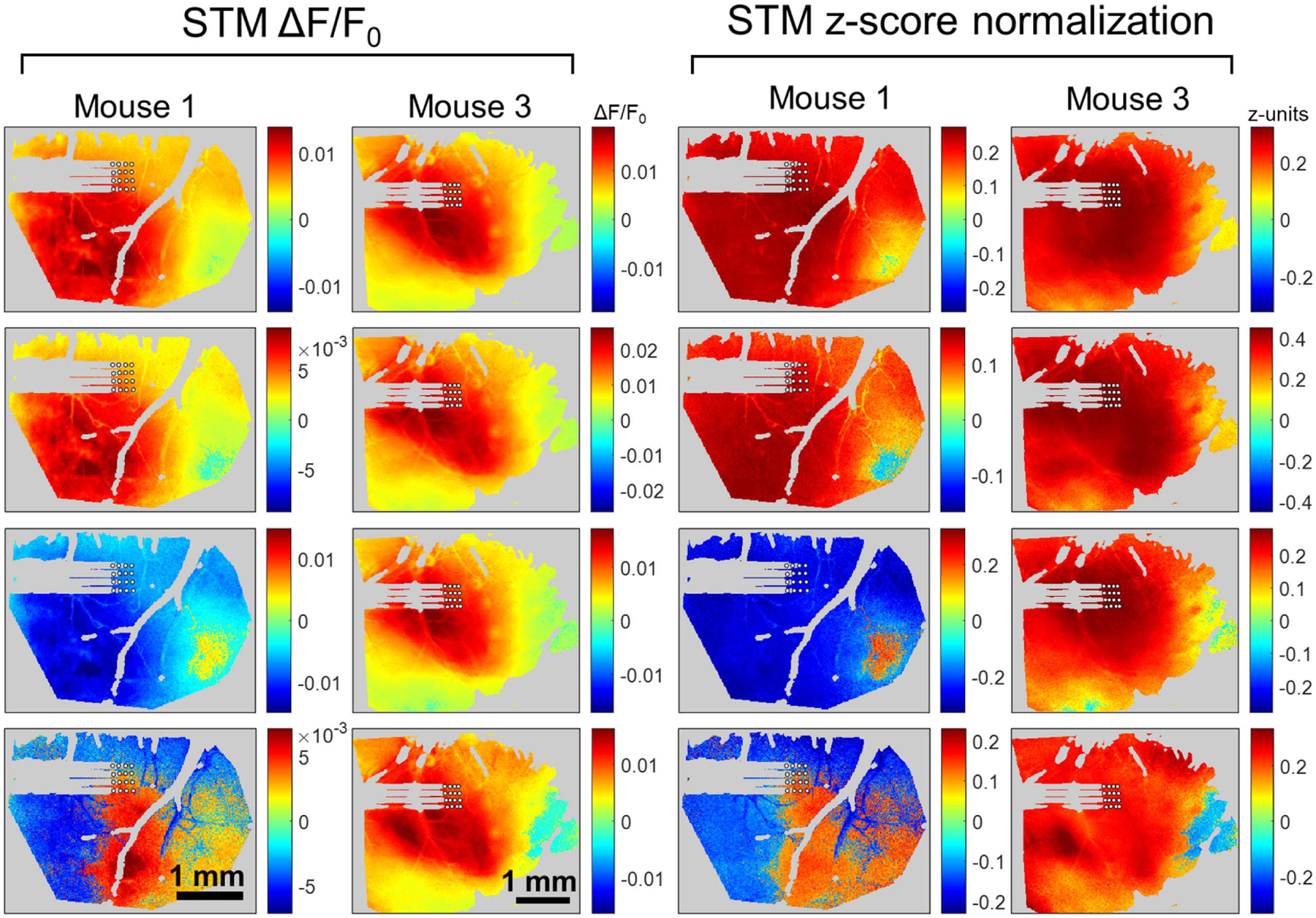
Comparison of four spike-triggered maps calculated using ∆*F*/*F*_0_ (left) and using *z*-score normalization (right.) Each pixel of the spike-triggered average map is the highest absolute value of the spike-triggered response with −1 to 1 s of the spike time. Red regions of the image indicate areas of increased activation and blue regions represent areas of inhibition in the spike-triggered response. STMs obtained with ∆*F*/*F*_0_ normalization tend to share a similar shape in the areas of highest activation or inactivation, which corresponds to the area of highest variability in the calcium imaging for that animal. By employing *z*-score normalization, more diversity in the STMs of different units is uncovered.

Because the regions of greatest variation in the GCaMP signal appear to dominate the spike-triggered responses, *z*-score normalization was employed before STA to better investigate the connectivity of each neuron. Figure 8 displays the effect of *z*-score normalization on the STMs for the four units used in figure 7. In both mice, STMs highly resemble the areas of highest overall GCaMP signal (figures 7(a) and (d)) With the application of *z*-score normalization, these STMs deviate from those regions of greatest activation, shown on the right of figure 8. These maps reveal a wider area of activation associated with each unit. That is, regions with relatively low magnitude of activity still show a response that is temporally associated with spiking, but this is not apparent in the STMs without *z*-score normalization. The *z*-score STMs also reveal more diversity in the responses of the different units. In some cases, the most activation is seen in the immediate vicinity of the electrode. In others, an area some distance from the electrode is revealed to be associated with a response to that neuron.

**Figure 9.**
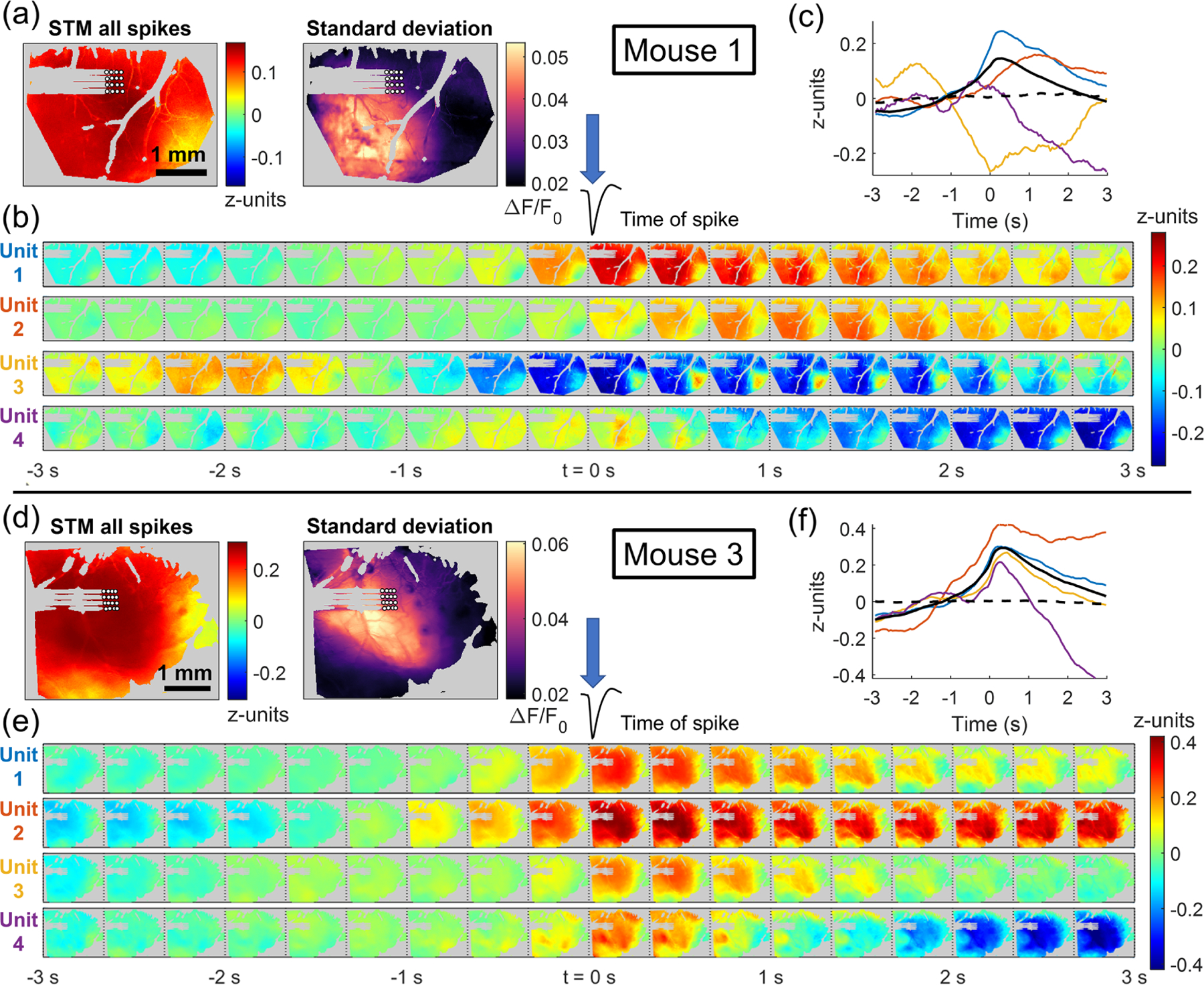
Example of spike-triggered average response for four spiking units from mouse 1, day 28 (top) and mouse 3, day 14 (bottom) obtained using *z*-score normalization of the calcium signal. (a) and (d) Comparison of spike-triggered map (STM) for all spikes and standard deviation of the time course of each pixel. (b) and (e) Example spike-triggered average responses from −3 to 3 s around the time of the spike. (c) and (f) Average time course of the spike-triggered responses within an ROI chosen from an area of high activation on the standard deviation map. The solid black line represents the combined spike-trigged response of all spikes from all channels. The dashed black line represents the spike-trigged response from randomly chosen spike times. The spike-triggered responses here are no longer dominated by the regions of highest variability as in figure 7, and more diversity between different units is observed.

Figure 9 reproduces the STA responses shown in figure 7 but obtained with *z*-score normalization. Spatially, the STA responses display the trends discussed above: wider area of activation or inhibition and increased diversity between units. The average time course of the responses, shown in figures 9(c) and (f), are largely unchanged, as the *z*-score normalization only affects the relative magnitude of changes in different pixels and does not change the shape of the response associated with a spiking neuron.

**Figure 10.**
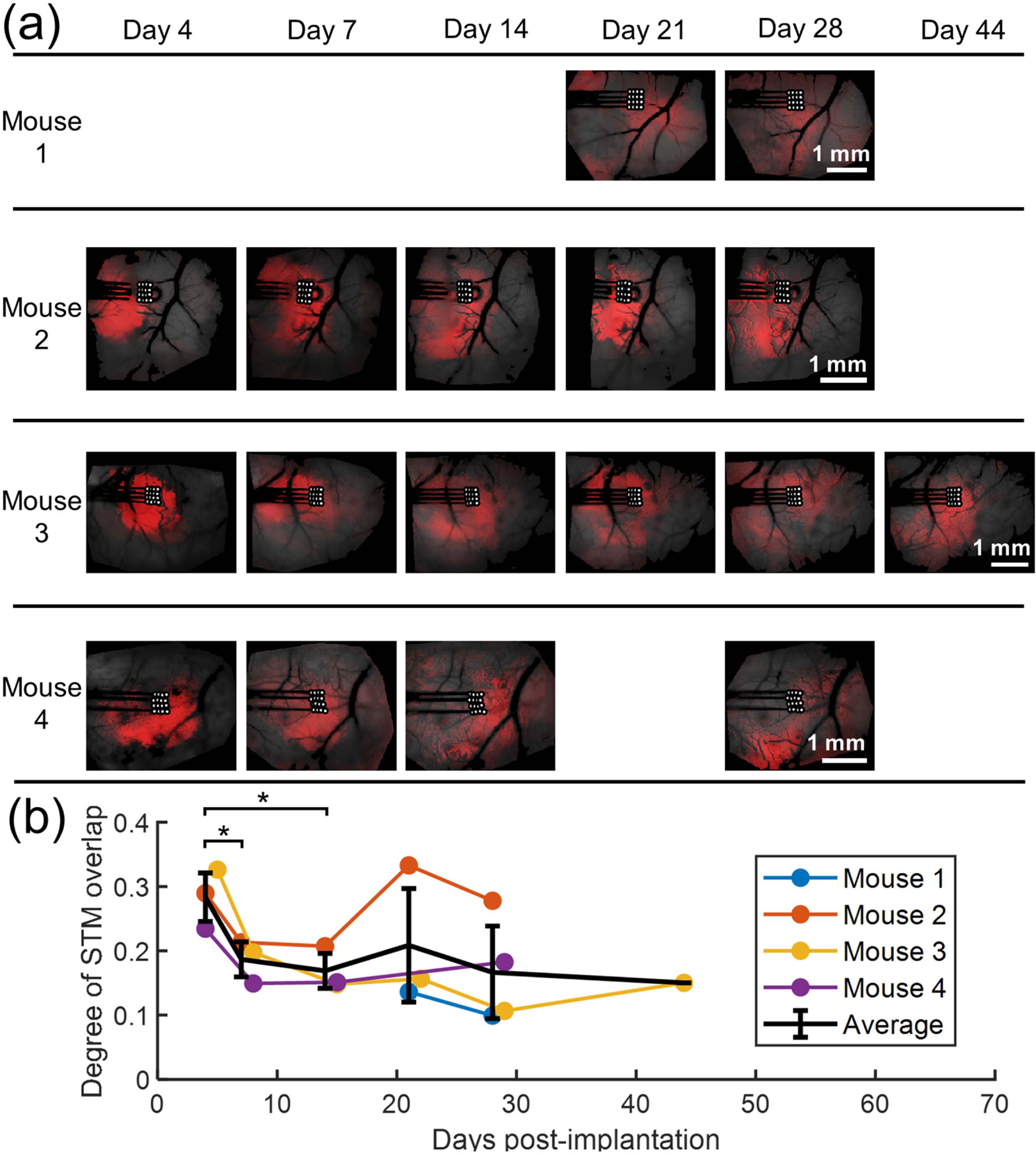
(a) Heatmaps summarizing STMs for all units of each measurement. STMs are thresholded to the 85th percentile and red intensity indicates the proportion of units whose thresholded STM includes that pixel. (b) Degree of STM overlap (mean dice coefficient between pairs of STMs) over time. Error bars represent the standard error of the mean. Asterisks indicate statistical significance with *p <* 0.05.

To analyze the similarity of the STMs within each measurement, STMs were thresholded to the 85th percentile and heatmaps of the thresholded STMs are displayed in figure 10(a). Red intensity corresponds to the number of units whose thresholded STM includes that pixel. Overlap between each pair of STMs is then calculated via the dice similarity coefficient and arranged into a dice coefficient matrix. The mean of the upper triangle of this matrix is defined as the ‘degree of STM overlap,’ and the result is shown in figure 10(b). There appears to be a trend towards high similarity between STMs at day 4, and more diffuse response at later time points. This is consistent with increased neuronal synchrony shortly after injury. In a two-sample *t*-test, the difference in STM similarity between day 4 and day 7 is statistically significant with *p* = 0.042. For the comparison of day 4 with day 14, *p* = 0.25.

## 4. Discussion

In this study, we used simultaneous electrophysiology and widefield calcium imaging of pyramidal neurons in awake mice. We characterized the relationship between the two measurements and how that relationship changed over the weeks following probe implantation.

In all trials, a robust correlation was observed between MUA and GCaMP signal with an average correlation coefficient of 0.35. Cross-correlation between raw MUA and GCaMP revealed a time delay between spiking and fluorescent signal of approximately 0.3 s, consistent with the sensor’s kinetics. The magnitude of this correlation is comparable to that reported by Murphy *et al*, though with a shorter lag time, likely attributable to the faster kinetics of GCaMP6s compared with GCaMP3 [21]. After MUAs were convolved with a gamma kernel intended to mimic the GCaMP kinetics, the correlation magnitude increased to an average value of 0.67. The spatial dependence of the relationship between electrophysiology and the fluorescence signal was examined using both correlation maps and STA. When examining correlation maps, we found the MUA-GCaMP correlation maps and GCaMP intrinsic correlation maps to be very similar to each other, consistent with previously published results [21, 22]. Spike-trigged average maps showed distinctly different spatial patterns for different units when *z*-score normalization was employed. These STMs appear to indicate increased neuronal synchrony shortly after implantation, likely related to the acute injury response and early metabolic stress. This is visible in the bright red regions of figure 10 for early time points and more diffuse red areas at later time points.

We initially hypothesized that the region of highest correlation magnitude between MUA and GCaMP would be the region over the recording site, but instead found large areas of high correlation with the peak correlation sometimes located a significant distance from the recording site (0.5–1 mm), figure 6. This result is partially explained by correlation maps of intrinsic correlation within the GCaMP image. We consider the GCaMP self-correlation maps to represent a limit on spatial discrimination in the MUA-GCaMP correlation maps. That is, when regions of the cortex experience activity that is highly correlated within that region, correlation with MUA will also exhibit a high correlation with that entire region. In one animal (Mouse 1) we observed different spatial patterns in the correlation maps of different channels as well as high correlations occurring at the greatest distance from the probe. This is evidenced in the diffuse red areas in the top row of figure 6. Murphy *et al* [21] report only one example of an MUA-GCaMP correlation map, in which two large cortical regions display high correlation: localized to the recording site as well as the corresponding area of the contralateral hemisphere. Clancy *et al* [22] report single-unit correlation maps and show that some units exhibit the highest correlation with cortex regions very close to the recording site, and other units display higher correlation with a region far from the recording site. Our results appear to be in line with those two studies, as a high correlation with calcium imaging is observed relatively close to the recording site (∼0.5–1 mm) Our relatively small field of view is a limitation of this study, as it is unknown if correlation would also be present in the contralateral hemisphere and reported by both Clancy *et al* [22] and Muphy *et al* [21].

When examining 2D correlation maps, we observed strong similarities between the MUA-GCaMP correlation maps and GCaMP intrinsic correlation maps. This is consistent with the results of Clancy *et al*, who also report some units with highest correlation co-localized with the recording site and other units with the highest correlation in a different region [22]. In that work, the recording was performed only hours after probe implantation, likely before the peak injury response had occurred. Here, we record over a longer period of time and observe correlations similar to the locally correlated neurons in that study.

When STA is performed on the GCaMP data without *z*-score normalization, figure 7, results are consistent with the results of Xiao *et al* [23] for cortical neurons in that neurons within the same imaging session yield nearly identical STMs. It was noted that these highly consistent STMs appeared to be dominated by the regions of the image with highest variability in fluorescence. That is, the regions with higher variance overall were found to have the highest change in spike-triggered fluorescence. *Z*-score normalization of the GCaMP data was used to control for the overall degree of variance and determine instead which pixels display activity that is independently associated with a given single unit, figure 9. Using this method, distinct STMs are obtained for different neurons within the same recording, which we believe to more accurately reflect differing projections of cortical neurons. These maps yield additional information over the MUA-derived correlation maps. In particular, many single units yield distinct STMs within a single recording site. Based on the time-course of spike-triggered response, some single units appear to consist of inhibitory neurons, such as Unit 3 in figure 9(c).

Though more data is needed to draw a strong conclusion, the results of this study suggest STMs obtained at the earliest time point show more consistency between neurons while STMs from later time points show more diversity. Heatmaps of the thresholded STMs showing these differences over time are shown in figure 10(a). As increased neuronal synchrony has been noted during the injury response [1], as well as a decrease in inhibitory tone [16, 31], this synchrony could be responsible for the consistency of the STMs between units. Taken together with the correlation maps, these results suggest that during conditions of high neuronal synchrony, neither method can be used to spatially separate differences in the activity of different cells. While the STA analysis identifies regions that are statistically likely to follow spiking a given neuron, future work should compare the STM results with the response generated from neurostimulation, to determine if results exhibit less variation in triggered response at the earlier days.

Effects of the brain’s injury response are also evident in the widefield GCaMP signal alone. *K*-means clustering of pixels based on correlation was used to group regions of similar neural activity. Judged visually, this clustering is somewhat consistent between later time points but displays the most difference at the early time points after implantation. When the relationship between these clusters is compared to the reference day, FCS to the reference day starts lower at early time points and then trends upward. This indicates that functional connectivity is initially perturbed following injury but then stabilizes to a consistent relationship. However, it is not possible to determine from this study if the final functional connectivity relationship returns to the original state present before surgery or not. It was not feasible to compare function connectivity directly to the day 0 (pre-implantation) data due to the effects of anesthesia on that time point.

Recording quality metrics SNR and spike-yield did not significantly decrease over the time period of this study, as shown in figure 4. Though impedance was not measured directly here, these results may indicate that there was not a large increase in impedance between electrode and cortex, as increased impedance is associated with a reduction in signal quality, though the relationship is complex [1]. The firing rate and coefficient of variation of spike trains also did not significantly change over the study period.

Results in the literature are mixed as to the time frame in which recording quality degrades in intracortical microelectrodes, with some showing SNR remaining fairly stable up to four or six weeks. This is especially when SNR is calculated only for site which continue to record spikes, as is done here [32–34]. Additionally, studies which directly measure impedance differ in what kind of impedance increase is observed within four to six weeks [2, 11, 13, 33, 35]. Thus, we conclude that the lack of significant degradation of recording quality over this study is within the range of results reported in the current literature [36].

While spike yield does not significantly decrease over time, there may be a correlation between day-to-day variations in spike yield and the level of nearby brain activity. While the widefield calcium imaging used here cannot discriminate only cells within the detection range of the recording sites, these results suggest that reduced spike yields in this time frame may be related to a reduction in brain activity in the vicinity of the probe. More research is needed over a longer period to determine if the degrading signal quality of chronic implants is correlated with a reduction in calcium activity.

## 5. Conclusion

Concurrent electrophysiology and widefield calcium imaging yields a robust correlation between the two modalities, which remains consistent over the study period. Great overlap is observed between MUA-GCaMP correlation and intrinsic GCaMP correlation with the recording site region. Both correlation maps and STA show increased synchrony of the brain during the initial phases of the injury response. In the later phases, STA using *z*-score normalization of the calcium signal can be used to visualize diverse spatial responses between cells. Further study of simultaneous electrophysiology and widefield calcium over a longer time post-implantation, combined with impedance and explant analysis, would be valuable in assessing whether changes in signal quality are due to changes in local brain activity or due to electrode failure.

## Supplementary Material

supplemental figure

Supplementary material for this article is available online

## Data Availability

The data that support the findings of this study are openly available at the following URL/DOI: https://doi.org/10.6084/m9.figshare.27941697.
