# Supplementary material for: Longitudinal changes in electrophysiology and widefield calcium imaging following electrode implantation
Source: J Neural Eng. Author manuscript; Available in PMC 2026 Mar 11. (PMC12976863; doi:10.1088/1741-2552/ada0eb)
Supplement: supplemental figure [file NIHMS2134881-supplement-supplemental_figure.pdf]

Longitudinal changes in electrophysiology and widefield calcium imaging following electrode implantation

Constance Robbins[1], James Eles[2], X. Sally Zheng[2], Takashi Kozai[2], X. Tracy Cui[2,3,4], Alberto Vazquez[1,2]

[1]Univeristy of Pittsburgh, Department of Radiology, 203 Lothrop St, EEI Suite 700, Pittsburgh, PA 15213
[2]University of Pittsburgh, Department of Bioengineering, 302 Benedum Hall, 3700 O'Hara St, Pittsburgh, PA 15260
[3]Center for Neural Basis of Cognition, University of Pittsburgh and Carnegie Mellon University, Pittsburgh, PA 15260
[4] McGowan Institute for Regenerative Medicine, University of Pittsburgh, Pittsburgh, PA 15260

Supplementary material

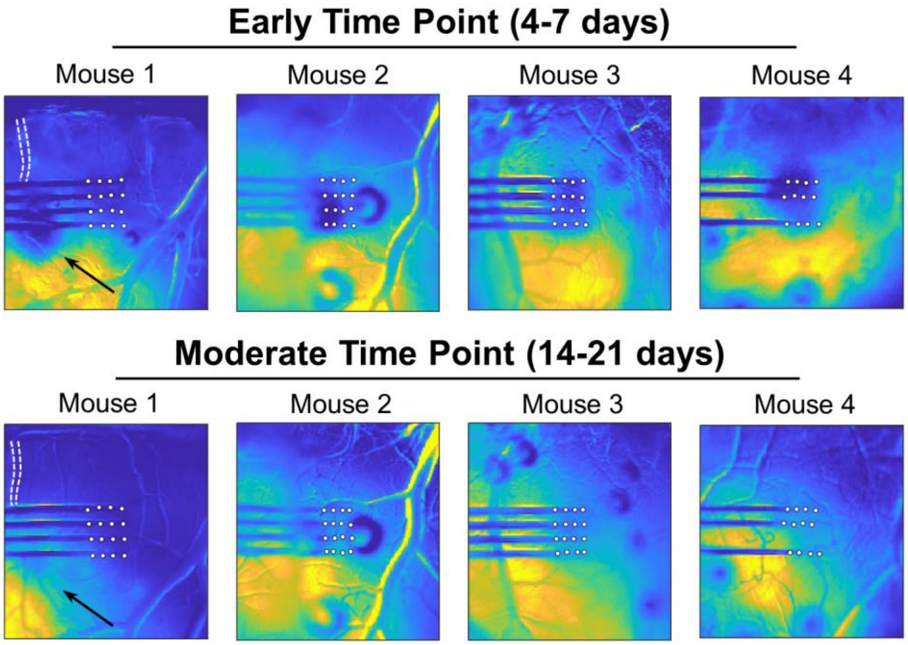

Figure 1S Examples of maps of power spectral density within the frequency band 0.1-1.2 Hz for four animals. Three animals show reduced activity in the vicinity of the recording sites at early time points which recovers at later time points. One animal (mouse 1) shows a region of decreased activity located over the shanks of the probe (black arrow) rather than the shank tips. This response may be due to bleeding as it occurs near where the shanks cross near a large blood vessel (white dotted line.)
